# Frequency, severity and risk indicators of retinopathy in patients with diabetes screened by fundus photographs: a study from primary health care

**Published:** 2014

**Authors:** Saleh Memon, Shahid Ahsan, Qamar Riaz, Abdul Basit, Sikandar Ali Sheikh, Asher Fawwad, A Samad Shera

**Affiliations:** 1 Saleh Memon, FRCS, Director Projects, Community Based Projects, Al Ibrahim Eye Hospital, ISRA postgraduate Institute of Ophthalmology, Gadap Town, Malir Karachi-75040, Pakistan; 2 Shahid Ahsan, MBBS, MPhil, Assistant Professor, Dept. of Biochemistry, Hamdard College of Medicine and Dentistry, Hamdard University, Karachi, Pakistan.; 3 Qamar Riaz, MBBS, M.Sc , Course Supervisor, ISRA School of Optometry, Al Ibrahim Eye Hospital, ISRA postgraduate Institute of Ophthalmology, Gadap Town, Malir Karachi-75040, Pakistan; 4 Abdul Basit, FRCP, Professor of Medicine, Department of Medicine, Baqai Institute of Diabetology and Endocrinology, Baqai Medical University, Plot No. 1-2, II-B, Nazimabad No2, Karachi-74600, Pakistan.; 5 Sikandar Ali Sheikh, M.A ,Diploma in Diabetes Education, Project Manager, Community Based Projects, Al Ibrahim Eye Hospital, ISRA postgraduate Institute of Ophthalmology, Gadap Town, Malir Karachi-75040, Pakistan; 6 Asher Fawwad, M.Phil, Assistant Professor, Research Department, Baqai Institute of Diabetology and Endocrinology, Baqai Medical University, Plot No. 1-2, II-B, Nazimabad No2, Karachi-74600, Pakistan.; 7 A Samad Shera, FRCP, Director WHO Collaborating Centre, Diabetic Association of Pakistan, 5-E / 3, Nazimabad, Karachi-74600, Pakistan.

**Keywords:** Diabetic retinopathy, Fundus camera, Primary care diabetes centre

## Abstract

***Objective:*** To determine the frequency, severity and risk indicators of diabetic retinopathy (DR) in patients with diabetes attending a primary care diabetes centre.

***Methods:*** This observational study was conducted at Diabetic Association of Pakistan - a World Health Organization collaborating center in Karachi, from March 2009 to December 2011. Registered patients with diabetes were screened by two field fundus photographs. Retina specialists graded the signs of retinopathy according to diabetic retinopathy disease severity scale.

***Results:*** Of total registered diabetic patients (n=11,158), 10,768 (96.5 %) were screened for DR. Overall DR was found in 2661 (24.7%) patients. DR was found in decreasing order of frequency in patients with type 2 (n= 2555, 23.7%) followed by patients with type 1 diabetes (n=101, 0.93% ) and patients with gestational diabetes mellitus (GDM) (n=5, 0.46%). Among patients with DR, signs of non-sight threatening retinopathy was dominant. Females and patients of working age group predominantly had retinopathy. Type 1 patients >16 years and type 2 patients < 5 years of history of diabetes had sign of retinopathy in increased frequency.

***Conclusion:*** Every forth patient with diabetes in this large cohort had signs of diabetic retinopathy. Females and patients in working age group predominantly had retinopathy. Type 2 patients with short while type 1 patients with long history of diabetes most frequently had DR. Dissemination of the present study findings may help in increasing the awareness of this serious complication of diabetes.

## INTRODUCTION

Prevalence of diabetes is increasing at an alarming rate all over the world.^1^ The low and middle-income countries in particular are the major contributors to the global burden of diabetes.^2^ It is anticipated that more than 80% of the world’s population with diabetes will come from the developing countries and by the year 2030 more than 60% of the world’s population with diabetes is expected to be living in Asia.^3^

Like other countries in Asia, Pakistan is also confronting a rapidly growing epidemic of diabetes. With a population of 180 million^4^, currently nearly 7 million people in the country have diabetes.^ 5^ This number is expected to increase immensely in the coming years. It is projected that by the year 2030 Pakistan will have nearly 13.8 million people with diabetes, approximately two fold rise in the current prevalence of diabetes.^5^

The increasing prevalence of diabetes leads to increasing incidence of diabetes complications.^6^ These complications when undetected or untreated will almost inevitably have an impact on the quality of life of a person and become a burden on the health care system and the community.^7^ Diabetic retinopathy (DR) is one of the chronic complications of diabetes and a leading cause of visual impairment and blindness among people of working age group.^8^ Apart from its effect on vision, retinopathy has social and economic consequences.^8^^,^^9^ With the expected rise in the prevalence of diabetes, the complications of diabetes like DR could become even more burdensome for the people and the country.^8^

Since sight loss due to retinopathy is avoidable, it is imperative that every effort should be made to lessen the burden of this diabetes complication. To achieve this goal it is vital to have information regarding current magnitude of the problem. Several investigators attempted to address this issue in Pakistan. Wide variations in the reported frequency of retinopathy from 15% to 58% across these studies were observed^10^^,^^11^ These variations in the frequency were attributed to a number of factors that limited external validity of these studies. Moreover, due to small sample size and use of less sensitive screening modalities in most of these studies, reported frequencies may not be truly regarded as the actual representative of the disease burden.

The present study was conducted to determine the frequency, severity and risk indicators of diabetic retinopathy by fundus photographs in a large cohort of patients with diabetes registered at a primary health care centre in Karachi, Pakistan.

## METHODS

This observational study was conducted at the diabetes centre of the Diabetic Association of Pakistan (DAP), a World Health Organization (WHO) collaborating center in Karachi, Pakistan. All registered patients attending the out patients department of the diabetes centre of DAP from March 2009 to October 2011 were included. Ethical approval for the study was taken from institutional review board of the institute.

After obtaining informed consent, data on demographic, anthropometric and clinical parameters were collected from each patient on a specially designed proforma. Past history of any eye surgery or laser treatment or use of any ophthalmic drug were taken from each patient.

***Image Acquisition: ***After checking best corrected visual acuity retinal screening was done by a fundus camera, Canon CR – 1. Screening was performed without instillation of mydriatic drops in a dark room after adaptation of pupil in the dark. Two 45^o^ retinal images, one centre to the optic disc and other centre to the macula of each eye were taken and stored in JPEG format by patient’s name with a unique patient’s identification number on the hard disk and a compact disc (CD).

***Image Reading: ***The images acquired at DAP were graded at Al-Ibrahim Eye Hospital (AIEH) Karachi, a tertiary are teaching hospital in Ophthalmology. Retina specialists evaluated the retinal photographs. All four photographs of a patient were graded. Presence of signs of retinopathy in any photographs was taken as sufficient evidence to be classifying the patients in retinopathy group. Patients with inconclusive photographs were called at DAP for ophthalmoscopy and bio microscopy. In still uncertain cases Fluorescein Fundus Angiography and Optical Coherence Tomography were performed. Findings of these tests were recorded in the respective soft and hard files of the patients.

***Grading of Diabetic Retinopathy: ***Signs of diabetic retinopathy were graded according to the diabetic retinopathy disease severity scale.^ 12^ Based on the severity of retinopathy lesions, patients with signs of diabetic retinopathy were categorized into non-sight threatening diabetic retinopathy (NSTDR) and sight threatening diabetic retinopathy (STDR) groups. Patients with mild and moderate non-proliferative diabetic retinopathy (NPDR) were categorized in NSTDR group. Whereas patients with proliferative diabetic retinopathy (PDR), clinically significant macular edema (CSME) alone or in combination with NPDR or PDR and ADED (advanced diabetic eye diseases) were included in the category of (STDR). Patients with severe non-proliferative diabetic retinopathy (SNPDR) were considered clinically on individual basis and decided either for follow up or treatment however, for the purpose of analysis this category was summed in the STDR.

***Statistical analysis*** *: *Data was entered and analyzed by Statistical package for Social Sciences (SPSS) version 13.0. All continuous variables were presented as mean ± standard deviation and categorical variables were expressed as frequency and percentage. Comparison between groups were done by ‘t’ test or chi-square test where appropriate for continuous and categorical variables respectively. P value < 0.05 was considered statistically significant.

## RESULTS

During the study period a total of 11,158 patients with diabetes were registered at the diabetes centre of DAP. Baseline characteristics of the study subjects are shown in Table-I. 

**Figure 1:**
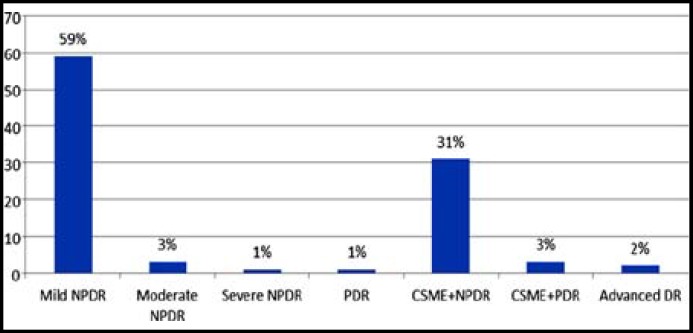
Frequency of DR severity in patients with type 1 diabetes

**Figure 2:**
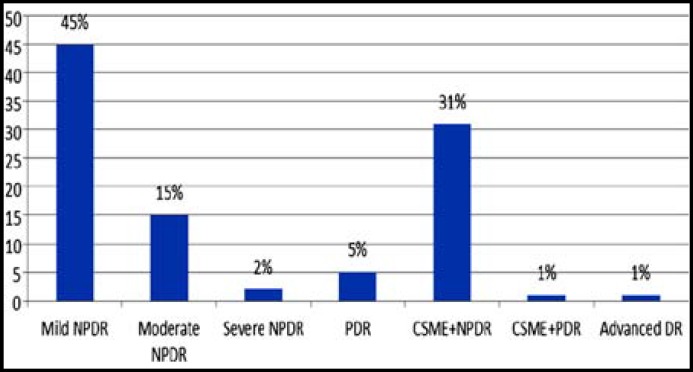
Frequency of DR severity in patients with type 2 diabetes

Of all the registered patients 96.5% (n=10,768) patients were screened for DR. Overall DR was found in 2661 (24.7%) patients. In decreasing frequency, DR was found in type 2 diabetes (n=2555; 23.7 %) followed by patients with type 1 diabetes (n=101, 0.93%) and patients with GDM (n=5, 0.46%). Irrespective of diabetes type (1 or 2), signs of NSTDR predominate among patients with retinopathy (Figure 1 & 2).

Study found significant difference in the mean age, BMI, waist circumference, SBP and DBP in patients with and without diabetes retinopathy of both type 1 and type 2 diabetes (Table-II).

Frequency distribution of the patient with and without retinopathy of both type 1 and type 2 diabetes is shown in Table-III. Signs of retinopathy were found in all age strata of both types 1 and type 2 diabetic patients. In increasing frequency DR was observed in 16-30 years in the patients with type 1 and 46-60 years age group in patients with type 2 diabetes. Regardless of diabetes type, retinopathy was found in increased frequency in females. Type 1 patients with >16 years and type 2 patients <5 years of history of diabetes had sign of retinopathy in increased frequency. Signs of retinopathy were also found in increased frequency in those having raised systolic or diastolic blood pressure in patients with type 1 or type 2 diabetes. 

## DISCUSSION

Prevalence of DR varies widely across different countries of the world^,^^10^^,^^11^^, ^^13^^,^^14^ Even studies originating from a country also corroborated varying frequencies of retinopathy.^10^^, ^^11^ In the present data over all, 24.7% of patients with diabetes had some form of retinopathy. Retinopathy was found in increased frequency in patients with type 2 diabetes followed by patients with type 1 diabetes. In least frequency retinopathy was found in patients with GDM. Diverse frequency of retinopathy in the present study, in patients with different types of diabetes substantiates the variability found in the literatures published nationally and internationally on the prevalence of retinopathy. A number of other factors besides type of diabetes were also attributed to this variation like community or hospital based study, use of different definition of diabetes and diabetes retinopathy and the use of different screening modality and the grading system.^10^^,^^11^^, ^^13^^,-^^15^ Thus direct comparisons of retinopathy prevalence across different studies are difficult. Frequency of retinopathy in the present study was higher compared to the reports from Australia and the West. ^8^^ ,^^13^^,^^14^. This decline in DR frequency can be attributed to the progress made by these countries to reduce the blindness incurred by diabetic retinopathy. Early disease diagnosis by mass awareness campaigns, centralized system of patient caring, screening and preventive treatment in these countries result in dramatic decline in the prevalence of diabetic complications.^15^ Increased frequency of retinopathy in the present study therefore demands the formulation and implementation of national diabetic retinopathy screening guidelines to protect the patients with diabetes from the expected increase in blindness incurred by retinopathy.

Diabetes is associated with long-term chronic complications. However the influence of age of onset of diabetes on the development of these complications is controversial. Some suggested early-onset diabetes is a more aggressive disease while other considered longer pre-pubertal duration of diabetes delayed the onset of diabetic complications.^16^^-^^18^ In the present study retinopathy was found in all age groups. More frequently it was found in younger age group (16-30 years) of patients with Type-1 diabetes, a finding consistent with the studies documenting increased incidence of retinopathy in post-pubertal age. It is speculated that puberty, characterized by both rapid growth, hormonal changes, and worsening in glycemic control, may accelerate the processes leading to chronic diabetes complications.^18^ Increased frequency of retinopathy among patients with type 2 diabetes was observed in the age group of 46-60 years compared to > 60 years of age. This may be due to varying expression of angiogenic growth factors with advance age in diabetes. These growth factors are implicated in the development of diabetic retinopathy but their angiogenic response to a stimulus is lessened in older versus younger individuals. Conceivably, ocular growth factors response to hypoxia and hyperglycemia may be greater in the younger patients, predisposing them to the development of retinopathy.^19^^,^^20^

**Table-I:** Baseline characteristics of the study participants (n=11,158).

	*Type 1* *n=656 (5.9%)*	*Type 2* *n=10414 (93.3%)*	*GDM* *n=88 (0.8%)*
Age (years)	25.73±12.2	53.02±10.59	30.54±3.72
Sex (M/F)	287 / 369 (43.8% / 56.3%)	3403 / 7011 (32.7% / 67.3%)	0 / 88 (0.0% /100.0%)
BMI	22.6±6.7	28.02±5.76	28.03±4.6
W.C (cm)	87.89±15.37	92.78±11.98	91.08±13.6
SBP (mmHg)	130.48±17.98	128.4±18.16	124.66±18.2
DBP(mmHg)	77.32±8.5	83.30±9.95	80.78±8.6
RBS(mg/dl)	249.45±94.42	237.27±97.65	211.4±101.76

Values expressed in (mean ±SD) or (n, %).

BMI: Body mass index, W.C: Waist circumference, SBP: Systolic blood pressure,

DBP: Diastolic blood pressure, RBS: Random blood sugar.

**Table-II:** Comparison of the patients with and without retinopathy

	*Type 1 (n=641)*			*Type 2 (n=10039)*		
	*Without retinopathy* *n=540 *	*With Retinopathy* *n=101 *	*p-value*	*Without retinopathy* *n=7484 *	*With Retinopathy* *n=2555*	*p-value*
Age(years)	24.59±11.68	31.14±13.28	0.000	52.16±10.55	54.06±9.61	0.000
Sex (M/F)	232 / 308 (43.0% / 57.0%)	45 / 56 (44.6% / 55.4%)	0.767	2322 / 5162 (31.0% / 69.0%)	947 / 1608 (37.1% / 62.9%)	0.000
BMI	22.16±6.48	24.96±7.20	0.000	27.98±5.67	28.22±5.94	0.066
W.C(cm)	88.32±15.21	84.74±16.47	0.035	93.11±11.99	91.52±11.61	0.000
SBP(mmHg)	128.48±15.04	141.53±26.74	0.000	125.40±14.99	137.29±22.94	0.000
DBP(mmHg)	76.94±8.57	79.50±7.98	0.006	83.11±9.88	83.88±10.12	0.001
RBS(mg/dl)	240.39±85.50	302.33±122.52	0.000	221.85±84.88	283.90±115.40	0.000

Values expressed in (n, %) or (mean ± SD).

BMI: Body mass index, W.C: Waist circumference, SBP: Systolic blood pressure,

DBP: Diastolic blood pressure, RBS: Random blood sugar.

**Table-III:** Frequency distribution of the patients with and without retinopathy

	*Type 1 Diabetes (n=641)*		*p-value*	*Type 2 Diabetes (n=10039)*		*p-value*
	*Without Diabetic Retinopathy* *(n=540)*	*With Diabetic Retinopathy* *(n=101)*		*Without Diabetic Retinopathy* *(n=7484)*	*With Diabetic Retinopathy* *(n=2555)*	
Age						
<15	102(18.9%)	5(5.0%)	0.000	4(0.1%)	0(0.0%)	0.000
16-30	321(59.4%)	58(57.4%)		194(2.6%)	27(1.1%)	
31-45	85(15.7%)	24(23.8%)		1759(23.5%)	451(17.7%)	
46-60	22(4.1%)	10(9.9%)		3932(52.5%)	1461(57.2%)	
>60	10(1.9%)	4(4.0%)		1595(21.3%)	616(24.1%)	
Gender						
Male (n %)	232(43.0%)	45(44.6%)	0.767	2322(31.0%)	947(37.1%)	0.000
Female(n%)	308(57.0%)	56(55.4%)		5162(69.0%)	1608(62.9%)	
	
Duration of DM						
<5	278(51.5%)	21(20.8%)	0.000	4138(55.3%)	1180(46.2%)	0.000
5-10	136(25.2%)	30(29.7%)		1875(25.1%)	611(23.9%)	
11-15	68(12.6%)	14(13.9%)		824(11.0%)	397(15.5%)	
>15	58(10.7%)	36(35.6%)		647(8.6%)	367(14.4%)	
		
Systolic Blood Pressure (mmHg)						
< 130mmHg	212 (39.3%)	28(27.7%)	0.028	3852(51.5%)	919(36.0%)	0.000
> 130mmHg	328(60.7%)	73(72.3%)		3632(48.5%)	1636(64.0%)	
Diastolic Blood Pressure (mmHg)						
< 85 mm Hg	468(86.7%)	85(84.2%)	0.501	4982(66.6%)	1595(62.4%)	0.000
> 85 mmHg	72(13.3%)	16(15.8%)		2502(33.4%)	960(37.6%)	
Random Blood Sugar (mg/dl)						
≤ 200	185(34.3%)	26(27.1%)	0.169	3420(45.7%)	773(30.8%)	0.000
> 200	355(65.7%)	70(72.9%)		4064(54.3%)	1733(69.2%)	

Data expressed as Mean ± Standard deviation and n (%). p-value < 0.05 considered statistically significant.

**Table-IV:** Projection for diabetic retinopathy in patients with type 1 and type 2 diabetes for country’s total population based on our study findings

*S. No*	*Type 1 diabetes*				*Type 2 diabetes*			
	*Components*	*Frequency*	*Projections*	*No of people (million)*	*Components*	*Frequency*	*Projections*	*No of people (million)*
1	Population <20 years	-	-	69.46	Population >20 years	-	-	59.71
2	Prevalence of type 1 diabetes	0.09%	0.09% of 1	0.06	Prevalence of type 2 diabetes	9.1%	9.1% of 59.51	5.43
3	Diabetic retinopathy	15.5%	15.5% of no 2	0.0096	Diabetic retinopathy	25.5% of no 2	25.5% of 5.43	1.38
4	NSTDR	62.8%	62.8% of no. 3	0.006	NSTDR	59.5% of no. 3	59.5% of 1.38	0.82
5	STDR	37.2%	37.2% of no. 3	0.0036	STDR	40.6% of no. 3	40.6% of 1.38	0.64

NSTDR: Non-sight threatening diabetic retinopathy, STDR: Sight threatening diabetic retinopathy.

Studies have consistently reported long history of diabetes, hypertension and degree of glycemia as the important determinants for the development and progression of diabetic retinopathy. In the present data, patients with type 2 diabetes having < 5 years history of diabetes, most frequently had retinopathy. It may be due to subtle symptoms of diabetes or inadvertent poor control of glycemia and during this period patients developed retinopathy. In contrast in patients with type 1 diabetes increased frequency of retinopathy was observed in those having > 16 years of history of diabetes. Plausibly it may be the result of obvious symptoms of diabetes in type 1 patients. Patients with type 1 diabetes seek medical treatment early in their course. Better glycemic control due to early diagnosis and treatment, spare these patients from developing retinopathy. Increased frequency of retinopathy in type 1patients with long history of diabetes (>16 years) and in type 2 patients with short history of disease (<5 years), also render the possibility of genetic influence in the development of retinopathy. It is anticipated that there are some factors harbored in the genes of these patients that result in either early or late expression of retinopathy. Thus potential role of genes should be sought in this population of patients.

Most of the patients with diabetes in the present study had hypertension and signs of retinopathy were increasingly found in those suffering from hypertension. Randomized controlled trial (RCT) and large cohort studies consistently showed an association between hypertension and the presence and severity of retinopathy in people with diabetes.^21^^,^^22^ Most of the patients in the present study had SBP a finding consistent with the UKPDS.^21^

Evidence from RCT^23^^,^^24^ and prospective study^25^; suggested glycemic control assist in reducing the development and progression of DR. Although in the present study, glycemic control of the participants were not assessed by estimating HbA1c nonetheless, estimation of random blood glucose depicted most of the patients with retinopathy had worse glycemic control. It therefore seems prudent to optimize blood glucose level irrespective of type of diabetes to curtail the rise in retinopathy prevalence. 

Present study has several distinct features compared to the earlier reports on retinopathy from the country.^,^^10^^, ^^11^ It is one of the largest studies ever reported on the frequency of retinopathy from Pakistan in which registered patients with diabetes (type 1, type 2 and gestational diabetes) were screened for the presence and severity of retinopathy. We used technologically advanced screening modality, a fundus camera, that has better sensitivity and specificity compared to conventionally used screening modality.^26^ Retinopathy was documented by evaluating two fields of retina having sensitivity comparable to that of gold standard seven field fundus photographs^26^and beyond, retinal photographs in the present study were graded by the retinal specialists.

Since, a large cohort of patients with diabetes in the present study was screened for retinopathy therefore; findings of the present study may be generalized to the whole country based on the existing data. This projection is based on 1998 census and thus has some limitations.^4^ However, it gives an estimate about burden of retinopathy encountered by the country (Table-IV).

## CONCLUSION

Every fourth patients in this large diabetic cohort had signs of retinopathy. Females and the patients of working age group more frequently had retinopathy. Findings of the present study may be regarded as the actual representation of the burden of retinopathy. Dissemination of the findings of the present study may help to increase the awareness of this serious complication of diabetes and to guide the population for early intervention to prevent further deterioration of vision.
